# IoT-Based Fish Farm Water Quality Monitoring System

**DOI:** 10.3390/s22176700

**Published:** 2022-09-05

**Authors:** Chiung-Hsing Chen, Yi-Chen Wu, Jia-Xiang Zhang, Ying-Hsiu Chen

**Affiliations:** 1 Department of Electronic Communication Engineering, National Kaohsiung University of Science and Technology, Kaohsiung 811213, Taiwan; 2 Microchip Technology Inc., Chandler, AZ 85224, USA

**Keywords:** aquaculture, wireless transmission, life expectancy, Internet of Things, robotic arm

## Abstract

Typhoons in summer and cold snaps during winter in Taiwan often cause huge aquaculture losses. Simultaneously, the lack of human resources is a problem. Therefore, we used wireless transmission technology with various sensors to transmit the temperature, pH value, dissolved oxygen, water level, and life expectancy of the sensor in the fish farm to the server. The integrated data are transmitted to mobile devices through the Internet of Things, enabling administrators to monitor the water quality in a fish farm through mobile devices. Because the current pH sensors cannot be submerged in the liquid for a long time for measurements, human resources and time are required to take the instrument to each fish farm for testing at a fixed time. Therefore, a robotic arm was developed to complete automatic measurement and maintenance actions. We designed this arm with a programmable logic controller, a single chip combined with a wireless transmission module, and an embedded system. This system is divided into control, measurement, server, and mobility. The intelligent measurement equipment designed in this study can work 24 h per day, which effectively reduces the losses caused by personnel, material resources, and data errors.

## 1. Introduction

Aquaculture is prosperous in the western coastal areas of Taiwan, and most of its traditional farming methods rely on human expertise. Whether it is the time control of the waterwheel aerator, feeding, or water quality testing, all require human resources and is time-consuming for farmers. The sudden cessation of aquaculture during cold snaps causes the farmers to suffer heavy losses. The water quality monitoring instruments that are easy to obtain, such as pH and DO meters, are mostly hand-held and have traditional functions. pH and DO must be manually measured and the meters cannot perform regular measurements, return data, or perform form rendering.

Therefore, solving the measurement problem is fundamental. pH sensors cannot be submerged in liquid for an extended time and must be immersed in protective liquid during non-measurement periods to ensure its accuracy and prolong its life. The solution we propose is to use a self-designed robotic arm equipped with a pH sensor to replace the human arm and to measure the water quality of the fish farm. The water quality data are submitted to a set database for comparison and to determine whether the water quality meets the living condition requirements of the fish. However, due to cost considerations, it is impossible to assign one set of equipment to each fish farm. Therefore, the solution is to place a submersible motor in each fish farm and extract the water. Then, the pH sensor of each fish farm sequentially measures and is maintained through a set of robotic arms. The original concept was presented at ISPACS 2021 [1].

Z. Shareef and S. R. N. Reddy developed a real-time water quality monitoring system with widely distributed wireless sensor elements that transmit data back to the main control terminal [2]. Judging the water quality status through data analysis, when the water quality is abnormal, a warning is sent to the farmers, so that preventive measures can be taken in time. However, this type of water quality monitoring system still needs widely distributed monitoring nodes in the fish farms, which will be a big problem for the supply of electricity and the maintenance of equipment. In addition, the sensor needs to be regularly calibrated to improve the sensing accuracy, and frequent equipment calibration requires considerable human resources.

Jen-Yung Lin developed a system that integrates various sensors, including dissolved oxygen, pH, and water temperature in each water layer [3]. This system uses Modbus TCP/IP communication to return the collected data and then provides the data to breeding managers through web data and mobile devices. The wired transmission of data can reduce the transmission problems caused by poor signal, but it limits the field size and increases the difficulty of setting. Management systems based on the Internet of Things and artificial intelligence are system architectures that many industrial blocks can use. Industry, commerce, agriculture, and fishery can all be improved through this architecture. The water productivity per unit on land is lower than the water productivity of the same volume at sea, which diminishes the economic benefits of aquaculture on land and leads to overfishing of wild resources, which has caused many conflicts between farmers and government. Through technological methods, traditional aquaculture has become a semiautomated or fully automated breeding system, which can effectively solve the conflicts between humans and the environment, ensure the livelihood of farmers, and help with achieving environmental protection sustainability. For the government, national breeding and environmental management can be directly checked through the Internet of Things and databases, which can save human resources. An auxiliary fishery breeding system designed based on the Internet of Things can become a truly fully automated breeding system. In addition to improving the output and quality of fish farms, it can also ensure the integrity of the natural environment through systematic monitoring technology to achieve the integrity of fishery resources and the sustainable development of the marine environment. Here, we use grouper to illustrate: the water temperature range is 22~28 °C, the pH range is 7~9, the dissolved oxygen content is 5 (mg/L), etc.

Because water quality sensors are placed in liquid for a long time, the chemical reactions of the water quality sensor are frequent. The reaction solution in the water quality sensor becomes exhausted, and the detection head becomes polluted, reducing the life of the water quality sensor. Fishers use manual measurements or rely on observation of fish movements and surrounding environments to judge the water quality of fish farms, requiring extensive human and material resources. We used a self-designed robotic arm to replace human measurement and maintenance. The measurement equipment can work continually. Its high reliability and stability are major advantages. The cleaning function of the robotic arm can prevent the sensor from being affected by excessive consumption of the reaction solution or environmental factors in the fish farm. The main water quality parameters detected are pH, dissolved oxygen, and temperature. The problem that the sensor cannot be immersed in liquid for a long time is solved using a robotic arm as an alternative solution. According to environmental changes, the system uploads data by a time setting [4]. Fishers can monitor the water quality of the fish farms at any time through mobile devices [5,6]. When the water quality is abnormal, the server sends a message to mobile devices to notify fishers to avoid the loss of fish.

## 2. System Design

**Figure 1:**
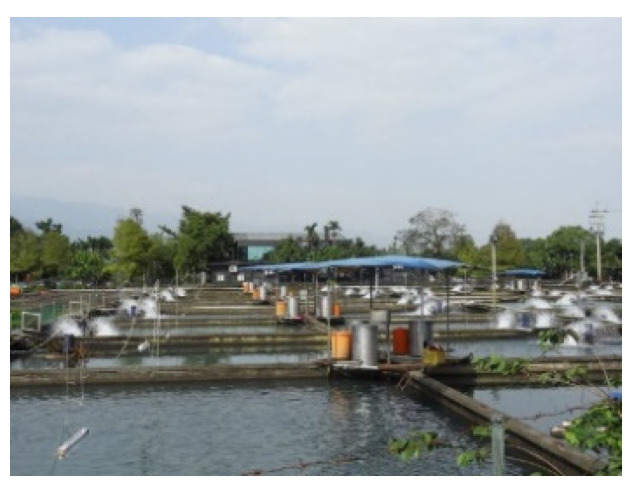
Fish farms in Taiwan.

**Figure 2:**
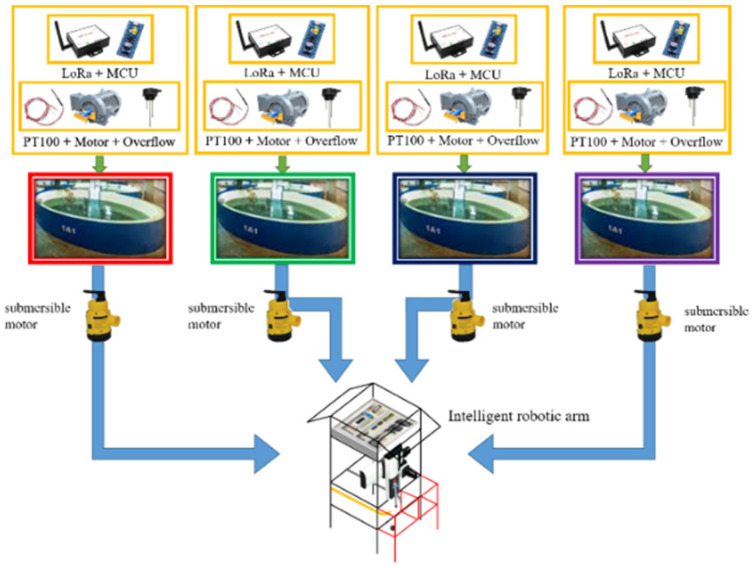
System architecture.

**Figure 3:**
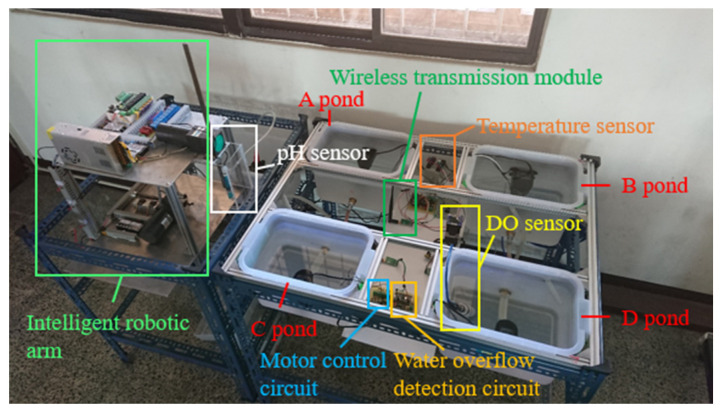
Simulation diagram of intelligent fish farm management system.

We integrated a programmable logic controller (PLC) with microprocessor signal processing and a PC-based server and used NB-IOT technology to transmit measurement data to the server. The main advantages of this system include lower power consumption, the ability to execute multiple threads at the same time, and improved processing performance. Figure 1 shows typical fish farms in Taiwan. This system design mainly simulates fish farms, as shown in Figure 2. Figure 3 is the simulation fish farm with four ponds. The ponds A to D in the figure are the simulated fish farms. Each pond uses a submersible motor to pump water to the measuring tank under the robotic arm, which then use the pH sensor mounted on the robotic arm to measure. After the measurement, the robotic arm automatically cleans the sensor head to prevent the accuracy of the pH sensor from being affected by the previous use. Each pond has a temperature, dissolved oxygen, and water overflow sensor. Each sensor sends data from the embedded system to the server through a wireless transmission module [7,8,9,10]. It is convenient to capture the data with mobile devices and web pages, so fishers can easily view the water quality status of each pond [11,12,13]. 

### 2.1. Robotic Arm Subsystem

**Figure 4:**
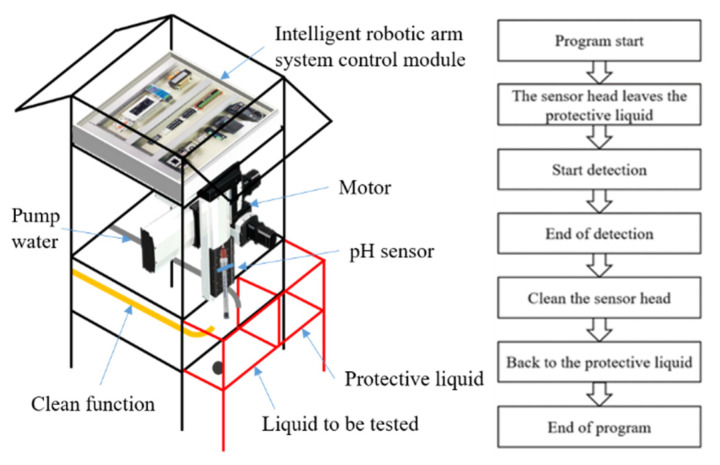
Intelligent measurement arm and its flow chart.

Because a pH sensor cannot be placed in liquid for a long time to measure, it takes humanpower and time to take the instrument to each pond for testing at a fixed time. The self-designed robotic arm as developed is shown in Figure 4. When the set time is up, a series of actions begins. The motor drives the rod connected with the pH sensor to make it leave the protective liquid, and then the rod drives the pH sensor down in the liquid to be tested. After the set time is elapsed, the pH sensor leaves the liquid to be tested and rises to a fixed point for cleaning. In this way, the robotic arm completes a series of measurement and maintenance actions.

### 2.2. Integrated Architecture

**Figure 5:**
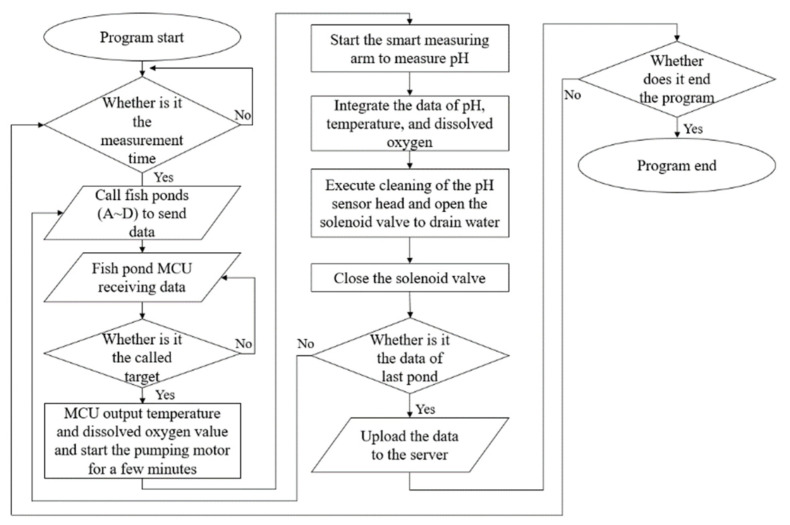
The flow chart of integrated architecture.

A flow chart of integrated architecture is shown in Figure 5. After receiving the signal from each pond, the system transmits the data on the water quality of the pond and starts the submersible motor to pump the liquid tested from each pond to the tank under the robotic arm to be measured by the pH sensor. After a series of measurements, the system judges whether testing if finished for all the ponds. Finally, the data are combined and sent back to the server via myRIO [14] for real-time monitoring on mobile devices [15,16,17].

### 2.3. Temperature Sensor Subsystem

Water temperature affects many properties: in addition to physical properties such as density, vapor pressure, and surface tension, it also affects chemical properties such as microbial growth, dissolved oxygen, and material response rate. Water temperature indirectly affects the measurement standards values of other indicators such as conductivity, dissolved oxygen, etc.

Most temperature sensors on the market now have a problem with temperature drift. To solve this problem, we adopted the constant voltage method and characteristics of the bridge circuit, and low-pass filters to filter out the temperature drift noise generated and then an amplifier to amplify the signal.

**Figure 6:**
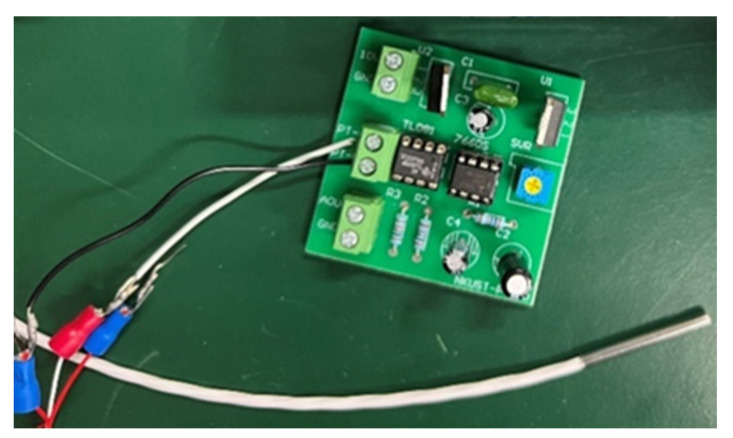
The Pt100 module.

In order to reduce the mortality rate, the water temperature of the growing environment of fish must be maintained. Pt100 was selected according to the environment and characteristics of the breeding pond, as shown in Figure 6. At the same time, it was necessary to avoid the expansion and contraction of the platinum wire body caused by temperature change because the error caused by temperature changes affects the measurement results.

### 2.4. Dissolved Oxygen (DO) Sensor Subsystem

**Figure 7:**
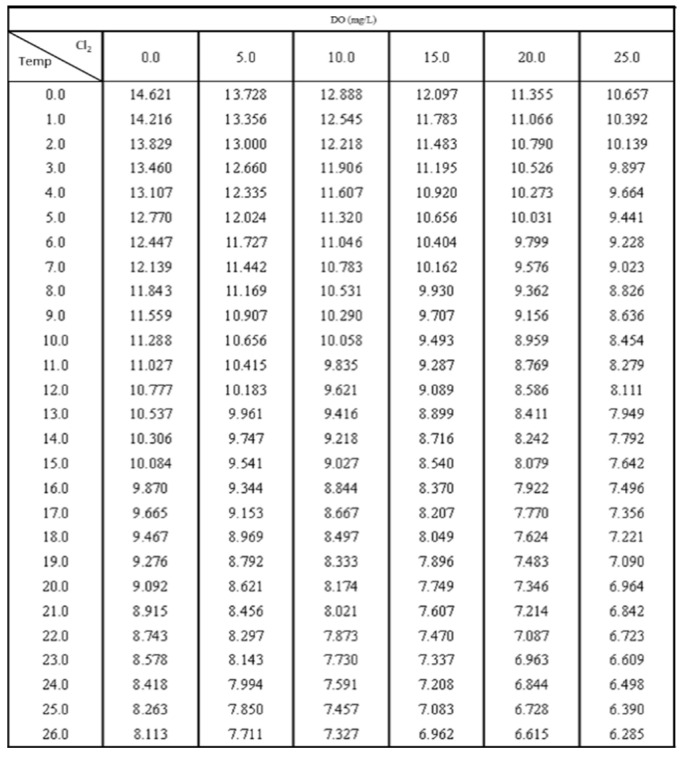
The relationship between temperature and saturated dissolved oxygen (mg/L).

Dissolved oxygen in water is one of the main indicators of water quality, representing the number of oxygen-consuming organisms in the water. When the water contains high concentrations of organic matter or nutrients such as phosphorus, it may be accompanied by high pH (acid-base value), and the list of saturated dissolved oxygen values in Figure 7 shows the relationship between temperature and saturated dissolved oxygen. The saturated dissolved oxygen in water with a water temperature of 25 °C and a chlorinity of 0.0 is 8.26 mg/L. It can be seen from the figure that the saturated dissolved oxygen degree in water changes according to the temperature. Therefore, temperature needs to be corrected when the percentage is used, and the temperature probe module used in this study already has a temperature compensation function [18,19].

**Figure 8:**
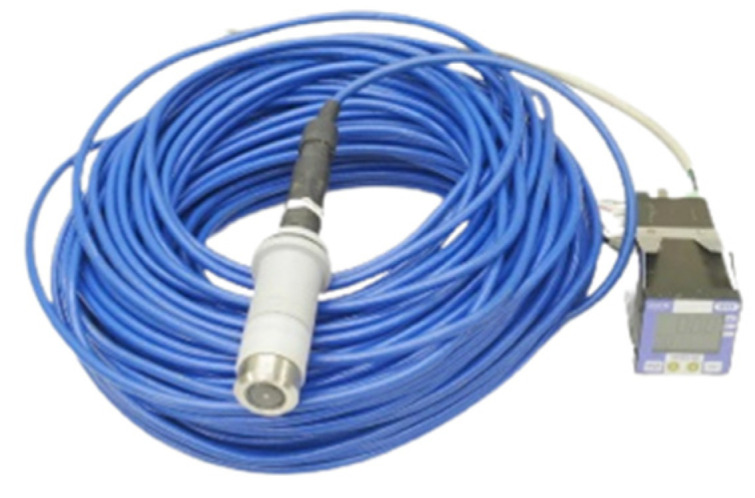
DO sensor.

Oxygen is the basic element required for biological survival. Therefore, in water quality management and aquaculture, dissolved oxygen is regarded as the primary indicator of water quality [20]. Generally, the higher the concentration, the better the water quality. Figure 8 shows the DO sensor used in this study.

### 2.5. Water Overflow Sensor Subsystem

**Figure 9:**
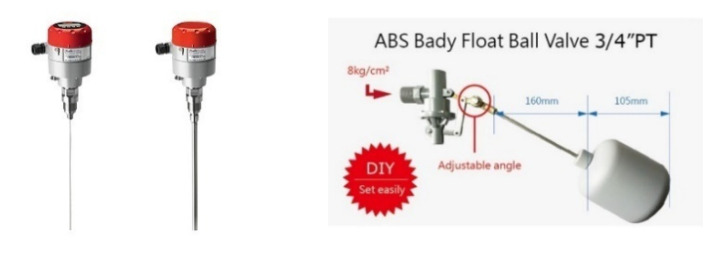
The traditional floating ball water level switch.

The water overflow sensor subsystem mainly replaces the traditional floating ball water level switch, as shown in Figure 9. In the traditional floating ball switch, the water level is mainly set at a fixed height, and the water level state cannot be accurately known. When there is a sudden rainstorm or the water quality needs to be adjusted, it may cause the fish to flow out. Therefore, the main water overflow sensor in this study identifies low, medium, and high water levels, and overflow, which is convenient for aquaculture so the water level can be adjusted according to the needs of farming. The principle of overflow primarily uses the characteristics of water conduction to judge water level. The advantage is that compared with the traditional float switch or the water overflow sensor in the industry, the system is more convenient and less expensive.

**Figure 10:**
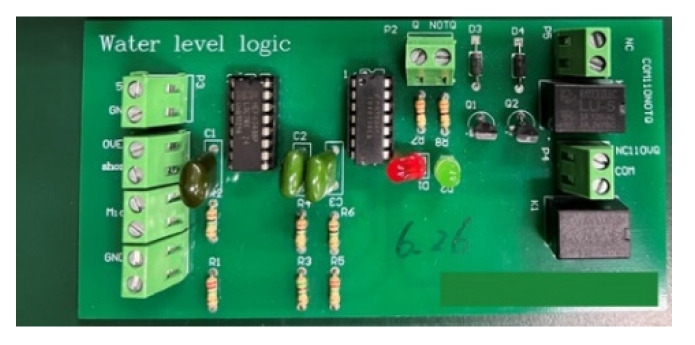
The module of the water overflow sensor.

The purpose of this subsystem circuit, as shown in Figure 10, is to adjust the water level within the safe height range [21]. When the water level is higher than the high-water level during typhoon season, the subsystem notifies the manager of the condition of the fish farms and reminds them to avoid loss of fish. When there is no rain for a long period of time, water level decreases due to evaporation. This decrease is not conducive to the survival of the fish. Therefore, an automatic pump motor is required to pump the underground reserve tank to replenish the water level to normal.

### 2.6. pH Sensor Subsystem

**Figure 11:**
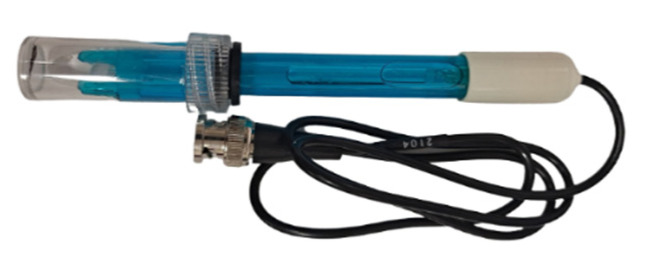
pH sensor.

The pH value, also known as the hydrogen ion concentration index, is a measure of the concentration of hydrogen ions in a solution. The large amount of excrement in fish farms produces amino acids, which increase the acidity of the water and cause the fish to become sick. The pH sensor used in this study is shown in Figure 11.

**Figure 12:**
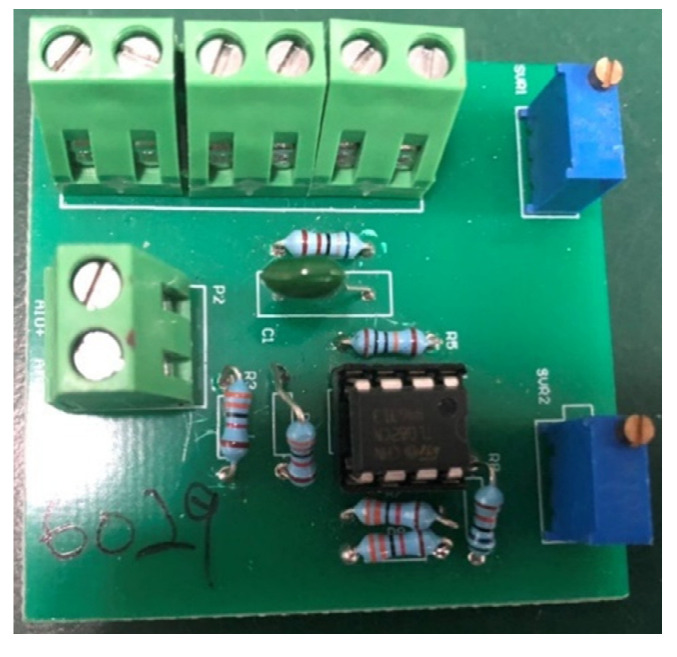
The pH module.

The required pH of the aquaculture environment for the growth of fish and shrimp is about 6.5~8.5. The survival rate of fish and shrimp in an acidic environment is higher than in an alkaline environment. Long-term water pH that is too high or too low makes the fish and shrimp sick, poisons them, stagnates their growth, and increases their mortality rates. After rainwater passes through substances such as carbon dioxide in the air, it becomes acidic. Therefore, when the weather is cloudy and rainy, the pH value of the pool water gradually drops due to the injection of too much rainwater. At this time, the body of water is acidic. In the case of long rainy days or heavy rain, the lack of sunlight causes many beneficial algae to die, and blue-green algae rapidly multiply. The density of algae plants in the water affects the pH value because the oxygen content in the water for photosynthesis by plants slowly rises during the day. Moreover, the pH value slowly rises at night because the pH value slowly decreases during respiration. Therefore, if the density of algae is too high or low, the dissolved oxygen in the water becomes insufficient, and the algae die and produce a large number of algal toxins. Furthermore, the excessive accumulation of fish and shrimp excrement or excessive feeding results in the excessive accumulation of waste at the bottom of the pool, which cannot be effectively decomposed, and the oxidation state efficiency significantly reduces. Continuous anaerobic fermentation produces a large number of anaerobic bacteria and toxic substances such as methane, hydrogen sulfide, ammonia nitrogen, nitrite, etc. In this study, to detect the life of the pH sensor and record the change in pH value, the detected data were compared with those obtained by the sensor. The specially designed pH sensing circuit is shown in Figure 12.

### 2.7. Low-Power, Long-Range Wireless Network LoRa System

LoRa is a physical layer or wireless modulation used to establish long-distance communication keys. Many traditional wireless systems use frequency shift keying (FSK), which can effectively meet the requirement for low power consumption. LoRa is based on chirp spread spectrum modulation, which retains the same low power consumption characteristics as FSK modulation but has a longer communication distance, higher network efficiency, and eliminates interference. LoRaWAN is used to define the communication protocol and system architecture of a network. It is a low-power wide area network standard launched by the LoRa Alliance, which can effectively realize the LoRa physical layer to support long-distance communication. This protocol and architecture have a profound impact on the terminal’s battery life, network capacity, service quality, security, and suitable application scenarios. In short, LoRaWAN is a wide-area network (WAN).

We used the LoRa star network architecture [22]. LoRa is a low-power, wide-area network (LPWAN) application, which mainly communicates with myRIO. The transmission distance exceeds 15 km and can handle up to one million nodes. Due to its low-power and long-distance advantages, the maximum data transfer rate is limited to 50 Kbps.

The LoRaWAN specification specifies the media access control (MAC) for the LPWAN [23]. LoRaWAN was implemented on top of the LoRa physical layer to specify the communication protocol and network architecture. These features greatly influence many performance parameters, including node battery life, network capacity, network security, and applications.

**Figure 13:**
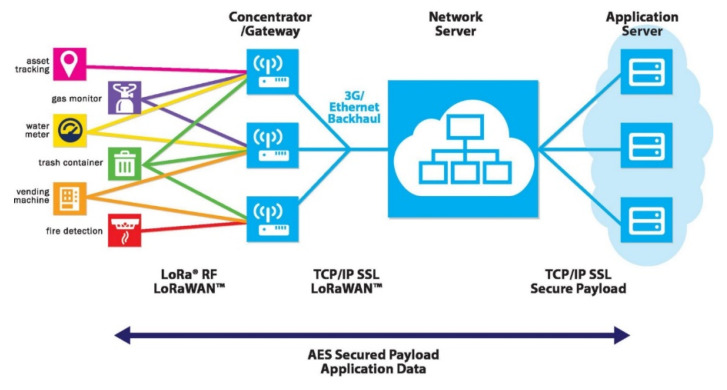
LoRa transmission module.

The LoRaWAN network architecture adopts a star-of-star topology, where each terminal node communicates with multiple gateways and then communicates with network servers, as shown in Figure 13.

### 2.8. Life Expectancy of Water Quality Sensor

Using sensors to analyze water quality replaces the previous method of judging the water quality through aquaculture expertise, which can improve water quality control and be used to detect early signs by observing changes in data, identifying problems and improving them. However, if the sensor rod is placed in water for an extended time, it becomes covered by biofilm, which affects the measurement accuracy and reduces the sensor’s life. Because most of the aquaculture is located on the coast, fishers use semifreshwater and semiseawater aquaculture types. The precise sensing rods are easily eroded by salt, so regular maintenance, replacement, or numerical correction is required.

**Figure 14:**
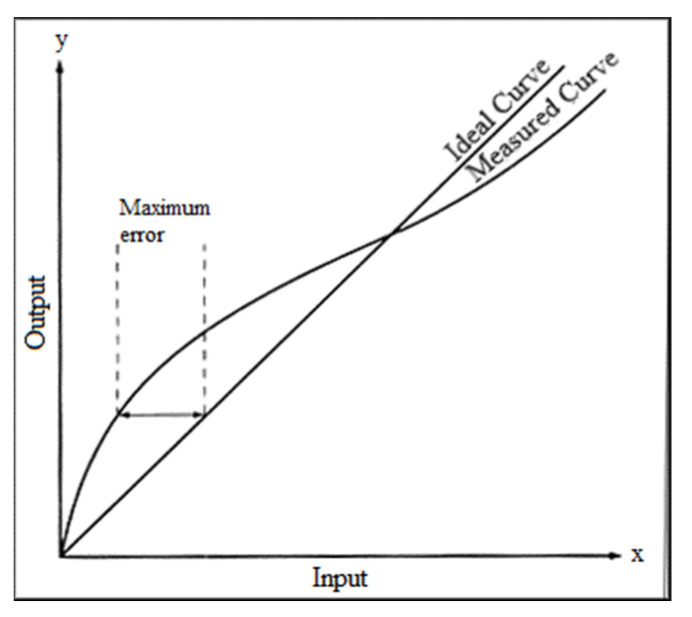
Characteristic curve offset graph.

**Figure 15:**
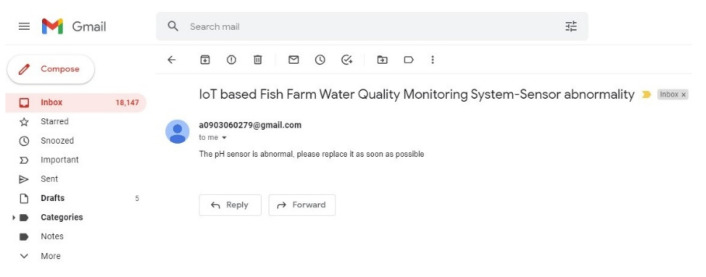
The alert e-mail.

When the life of the water quality sensor is about to end, its output characteristic curve can no longer maintain linearity, which means that we cannot make the measured value more correct through calibration, as shown in Figure 14. Therefore, a reliable detection method is needed to detect when the sensor needs to be replaced, a there is a life expectancy for the water quality sensor [24,25]. This detection first limits the sensor’s error percentage to 20% and then records the pH value measured before and after each calibration. Table 1 shows the pH value of the standard solution at each temperature [26]. The measured value before and after calibration is entered into the error calculation formula of Equation (1), and then the error values obtained each time are accumulated. When the error percentage reaches 20%, the current life is reset to zero. When the life of the water quality sensor reaches zero, the system displays this on the mobile terminal, and the server terminal and sends out an e-mail to remind the tester to replace the sensor, as shown in Figure 15. This way, additional losses caused by measurement errors are avoided.

(1) $$\%\mathrm{error}=\frac{\left|\mathrm{Exact}\;\mathrm{value}-\mathrm{Approximate}\;\mathrm{value}\right|}{\mathrm{Exact}\;\mathrm{value}}\times 100\%$$

## 3. Discussion and Conclusions

This system takes Arduino Mega 2560 as the core, which collects various water quality data and uses a programmable logic controller (PLC) to control the intelligent measurement arm’s machine and single-chip integrated motor control. It is equipped with an LPWAN expansion board for data transmission and monitoring of the entire breeding pond. It can display the data through the database of the terminal server and LabVIEW, which is easy for breeding managers to operate and does not require additional base station erection. Although the characteristics of using LPWAN include low power consumption, long distance transmission, low transmission volume, and low cost, LPWAN is not a solution and cannot replace all wireless transmission applications. For example, high-speed transmission requirements and instant applications need to rely on other transmission technologies.

Judging from the competitive situation of the three current LPWAN technologies, SIGFOX, LoRa, and NB-IoT, these technologies are good in terms of power consumption, transmission distance, and application fields, so it is difficult to say which will be replaced by which. These three technologies are more likely to be cross-applied according to different fields in the next few years, and the server will be set up on the cloud. Farm managers only need to connect to the LabVIEW window designed in this study, where they can connect to the Internet. The sensed data are graphed, and the remote monitoring of the breeding environment can reduce labor costs. NB-IoT is highly promising: the existing the network does not need to be rebuilt, and the industry chain is also consistent with the existing telecommunication network industry. However, for NB-IoT, a SIM card device needs to be installed at each node to develop the Internet of Things. This does not seem to be a practical business model if farmers have to pay telecommunications companies for equipment for each breeding pond and other equipment. Therefore, it is more likely that the communication protocol of LoRa and Wi-sun will be adopted in local area networks. After the data are uniformly transmitted to one point, they are transmitted together with NB-IoT and LTM to be more practical.

LoRaWAN can achieve data transfer rates of 300 bits/s to 50 kbit/s, process message payloads up to 243 bytes, and use a 433 or 915 MHz signal bandwidth. The network supports adaptive data transfer rates to maintain signal reliability under changing conditions. In this study, it reached a transmission distance of 4 km in the farming environment with a line-of-sight (LoS) range of up to 8 km. On the user side, distributed nodes can be developed and introduced into a commercial network, or a private network area can be established using their own LoRa area network.

**Figure 16:**
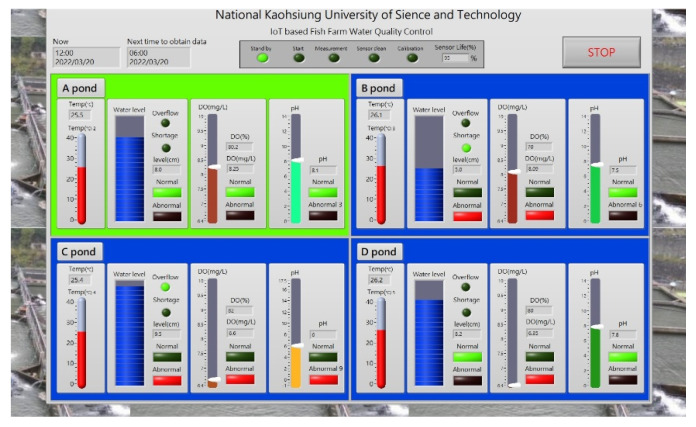
Human–machine interface of intelligent fish farm measurement system.

The measurement values are changing all the time, so the most important aspects are the real-time data transmission, design of the robotic arm, selection of materials, and installation tests. Improving the wireless transmission rate and durability of the equipment are also directions to be considered in the future. Figure 16 shows the human–machine interface of the intelligent fish farm measurement system. It shows the measured values of temperature, pH, water overflow, and DO in this study.

## 4. Future Outlooks

In the future, we aim to use the grouper model farm as a demonstration of smart farming and to test the effectiveness of the application of the proposed networked system, including water tank oxygen supply, feeding equipment, sensor control networking, feedback control, intelligent auxiliary decision-making, etc. We want to use big data to integrate various monitoring modules in breeding ponds in the field and initially establish a visual management system for the sensor control and networking of aquaculture facilities. This system will include subsystems such as breeding monitoring networking, activity monitoring, and breeding decision visualization of the intelligent environmental control facilities. Through the modular integration of various functions, including sensor control, networking, monitoring, and recording, the system will continuously collect key data such as water quality parameters, hydropower status, indoor and outdoor weather, and feeding and biological parameters during the breeding process, and will perform water quality analysis, bait feeding, water and electricity monitoring test verification, etc. The intelligent system will help with breeding decision-making, assist in control learning, and calculate the most appropriate decision-making control parameters, all of which will help to maintain the stability of the grouper model farm breeding environment and improve grouper breeding efficiency. 

In the future, artificial intelligence analysis and deep learning feedback control technology will be combined to build a complete intelligent breeding system including production technology, facilities, equipment, information, communication, Internet of Things, artificial intelligence, or related front-end technologies connected in series. We must continue to accumulate expert experience from water testing institutes and import the basic aquaculture environment parameters into database systems. To improve breeding, breeding experience can be accumulated, inherited, and adapted to form best practices.

## Figures and Tables

**Table 1:** The pH value of the standard solution at each temperature.

Temperature (°C)	pH 4.00	pH 7.00	pH 10.00
10	3.99	7.06	10.16
25	4	7	10
40	4.03	6.98	9.88
60	4.08	6.98	9.79

## Data Availability

Not applicable.
